# Lack of effects of fish oil supplementation for 12 weeks on resting metabolic rate and substrate oxidation in healthy young men: A randomized controlled trial

**DOI:** 10.1371/journal.pone.0172576

**Published:** 2017-02-17

**Authors:** Sebastian Jannas-Vela, Kaitlin Roke, Stephanie Boville, David M. Mutch, Lawrence L. Spriet

**Affiliations:** Department of Human Health and Nutritional Sciences, University of Guelph, Guelph, Ontario, Canada; Texas A&M University, UNITED STATES

## Abstract

Fish oil (FO) has been shown to have beneficial effects in the body via incorporation into the membranes of many tissues. It has been proposed that omega-3 fatty acids in FO may increase whole body resting metabolic rate (RMR) and fatty acid (FA) oxidation in human subjects, but the results to date are equivocal. The purpose of this study was to investigate the effects of a 12 week FO supplementation period on RMR and substrate oxidation, in comparison to an olive oil (OO) control group, in young healthy males (n = 26; 22.8 ± 2.6 yr). Subjects were matched for age, RMR, physical activity, VO_2max_ and body mass, and were randomly separated into a group supplemented with either OO (3 g/d) or FO containing 2 g/d eicosapentaenoic acid (EPA) and 1 g/d docosahexaenoic acid (DHA). Participants visited the lab for RMR and substrate oxidation measurements after an overnight fast (10–12 hr) at weeks 0, 6 and 12. Fasted blood samples were taken at baseline and after 12 weeks of supplementation. There were significant increases in the EPA (413%) and DHA (59%) levels in red blood cells after FO supplementation, with no change of these fatty acids in the OO group. RMR and substrate oxidation did not change after supplementation with OO or FO after 6 and 12 weeks. Since there was no effect of supplementation on metabolic measures, we pooled the two treatment groups to determine whether there was a seasonal effect on RMR and substrate oxidation. During the winter season, there was an increase in FA oxidation (36%) with a concomitant decrease (34%) in carbohydrate (CHO) oxidation (p < 0.01), with no change in RMR. These measures were unaffected during the summer season. In conclusion, FO supplementation had no effect on RMR and substrate oxidation in healthy young males. Resting FA oxidation was increased and CHO oxidation reduced over a 12 week period in the winter, with no change in RMR.

***Trial Registration***: ClinicalTrials.gov NCT02092649

## Introduction

Omega-3 polyunsaturated fatty acids are a family of fatty acids (FAs) characterized by unique physical and structural properties that influences several aspects of metabolism and physiology in the human body [1,2]. Increasing the intake of omega-3s exerts beneficial effects on multiple diseases with a metabolic and inflammatory component, including cardiovascular disease, obesity, and diabetes [3]. The shortest essential omega-3 FA is alpha-linolenic acid (ALA), which is necessary for the endogenous synthesis of the longer chain eicosapentanoic acid (EPA) and docosahexanoic acid (DHA). However, elongation and desaturation of ALA to EPA and DHA is not efficient in humans [4], meaning that EPA and DHA should be consumed in the diet.

One of the proposed mechanisms for the beneficial effects of omega-3 FAs is through incorporation into membrane phospholipids of different tissues, modifying the permeability and fluidity of the membrane, and influencing metabolic processes [5,6]. One tissue of particular interest is skeletal muscle which is metabolically active and comprises ~20% of the whole body resting metabolic rate (RMR) [7]. It has been hypothesized that a higher content of omega-3 FAs in skeletal muscle membranes increases whole body energy expenditure by altering the activity of membrane bound proteins, increasing mitochondrial proton leak [8], or enhancing protein synthesis [9]. For example, increased EPA and DHA in rat skeletal muscle membranes was associated with increased activity of the sodium potassium pump (Na^+^/K^+^) ATPase [8] and carnitine palmitoyltransferase I (CPT-1) [10], along with a decreased efficiency of the sarcoplasmic reticulum Ca^2+^ ATPase (SERCA) [11]. Furthermore, omega-3 FAs bind to peroxisome proliferator-activated nuclear receptors (PPARs), altering the expression of proteins involved in fat metabolism [12], such as fatty acid translocase/cluster of differentiation 36 [13], uncoupling protein-3 (UCP-3) [14] and CPT-1 [10]. Collectively these adaptations suggest that supplementation with EPA and DHA may increase whole body RMR and promote a shift towards fatty acid (FA) oxidation.

In healthy humans, evidence regarding the effects of omega-3 supplementation on whole body RMR and FA oxidation is limited and controversial. For example, some studies reported increased RMR (~4–5%) after fish oil (FO) supplementation [15,16], while others reported no effect [17,18]. Similarly, increased FA oxidation (22%) after FO supplementation has only been demonstrated in one study [15]. It is possible that the lack of consistency in the metabolic changes seen with FO supplementation was the result of biological and measurement variability, since the variance of consecutive RMR and substrate oxidation measurements has been reported to range between 2–5% and 15–25%, respectively [19,20]. Some factors that may have contributed to the variable results in previous studies include low doses of FO [15,17,18], shorter and variable supplementation periods [15,17], small numbers of participants [15,17], no control group [15,17], and lack of control for seasonal variation with metabolic measurements [15,16].

Therefore, the goal of this study was to investigate the effects of FO supplementation in healthy young adults on RMR and substrate oxidation, by utilizing a high dose of FO (2 g/d EPA, 1 g/d DHA), a long supplementation period (12 weeks), a significant number of participants (n = 13/group), use of a control group (olive oil), and controlling for seasonal metabolic changes.

## Materials and methods

### Subjects

Twenty-six healthy, recreationally active males (age 22.8 ± 2.6 yr; body mass 77.7 ± 8.7 kg; height 1.80 ± 0.07 m) who were involved in some form of physical activity 3–4 times per week volunteered to participate in this study. Written informed consent was received from each subject following a detailed explanation of the experimental protocol and any associated risks. Subjects were screened to ensure they were in good health (any medical condition or cardiorespiratory disease factor), and were excluded if they were currently taking (or had recently taken—past 3 months) omega-3 supplements or were consuming a diet high in omega-3s (ALA, EPA or DHA) more than twice a week. Subjects were instructed to maintain consistent dietary and exercise habits throughout the study, and physical activity records and three-day dietary records were obtained prior to and at the end of supplementation. The study was approved by the University of Guelph Research Ethics Board and by clinicaltrial.gov (#NCT02092649).

### Study design

**Fig 1:**
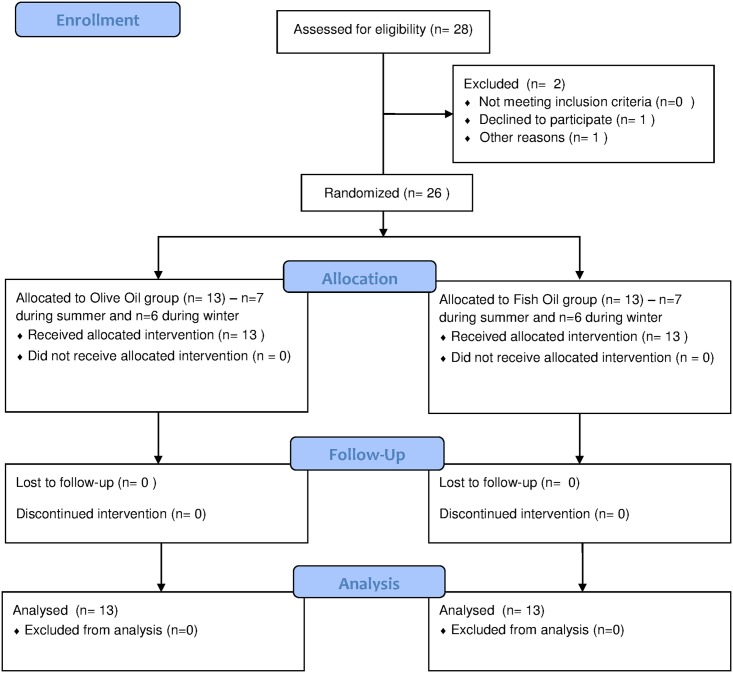
Consort figure illustrating participant flow through the study.

The study was conducted during two 12-week periods (Fig 1), where 14 of the 26 subjects were enrolled from the months of July-August until September-October (summer), and the remaining 12 from the months of October-November to December-January (winter). The monthly average temperatures during the summer were 17.6, 17.6, 14.1 and 9.2°C, and during the winter were 9.2, 0.1, -1.4 and -9.0°C. Subjects arrived to the laboratory (Department of Human Health and Nutritional Sciences—University of Guelph) to perform an incremental cycling test to exhaustion (VO_2max_) on a cycle ergometer. After 2–5 days, the participants performed a familiarization trial where RMR was measured following an overnight fast. Subsequently, subjects were matched for RMR, VO_2max_, age, body mass and blood parameters (Table 1), and were randomly assigned in a single blinded manner to either an olive oil (OO) or fish oil (FO) group. Pairs of subjects (one from each group) then visited the laboratory on two consecutive mornings at three different time points (weeks 0, 6 and 12) during the same time of the day for measurements of RMR and substrate oxidation. Fasted blood samples were taken (on the first day only) immediately after these measurements at weeks 0 and 12. A VO_2max_ test was performed 2–7 days after the last trial. Instructions for each trial were the same as the baseline trials.

**Table 1:** Subject characteristics and blood measurements.

	Olive Oil				Fish Oil			
	Week 0		Week 12		Week 0		Week 12	
	Mean	SD	Mean	SD	Mean	SD	Mean	SD
**Age (y)**	22	2	-	-	23	3	-	-
**Height (m)**	1.80	0.09	-	-	1.81	0.06	-	-
**Body mass (kg)**	77.8	10.3	78.5	10.4	77.5	7.1	77.6	6.9
**VO**_**2**_ **peak (ml/min)**	3457	563	3516	508	3495	538	3595	482
**Daily energy intake (kcal)**	2458	548	2337	525	2527	771	2506	687
**Triglycerides (mmol/L)**	0.83	0.17	0.81	0.29	0.84	0.35	0.68 *	0.20
**Glucose (mmol/L)**	4.60	0.33	4.49	0.36	4.64	0.39	4.62	0.30
**Cholesterol (mmol/L)**	4.31	0.66	4.25	0.51	4.18	1.10	4.02	0.68
**HDL-c (mmol/L)**	1.37	0.33	1.36	0.25	1.40	0.29	1.40	0.28
**LDL-c (mmol/L)**	2.57	0.61	2.53	0.58	2.40	0.99	2.29	0.64
**hsCRP (mg/L)**	1.08	0.81	0.89	0.83	0.71	0.52	1.12	1.05

All values are means ± SD.

*Significantly different (p = 0.04) from week 0.

### Supplementation

The OO group (n = 13) was supplemented with 3 capsules of Olive Oil (1 g olive oil per capsule, Swanson Health Products, Fargo, ND, USA) for a total of 3 g olive oil per day. The FO group (n = 13) was supplemented with 5 capsules of Omega-3 Complete (1 g fish oil per capsule, Jamieson Laboratories Ltd., Windsor, Ontario, Canada) per day, where each capsule provided 0.4 g of EPA and 0.2 g of DHA in triglyceride (TG) form, for a total of 2 g EPA and 1 g DHA per day. Subjects in both groups were encouraged to take capsules throughout the day (suggested to take 1–2 at breakfast and 2–3 at dinner). To promote supplement compliance, subjects were only given 2–3 weeks of capsules at a time. Written and oral reminders were also given periodically to ensure diet and exercise practices remained consistent throughout the study.

### Metabolic measurements

Subjects were asked to refrain from any physical activity, alcohol, and caffeine consumption 24 h prior to each study visit, as well as to consume the same diet the day before each resting metabolic and blood measurement. VO_2max_ was measured during an incremental exercise test to exhaustion (modified Astrand continuous test) on a cycle ergometer (Lode Sport Excalibur, Quinton Instruments, The Netherlands) using a metabolic cart (AEI MOXUS II Metabolic System, Pittsburgh, PA, USA). For the RMR measurements, subjects arrived at the laboratory (Summer relative humidity (RH): 44 ± 9%; T: 20.8 ± 0.8°C—Winter RH: 24 ± 10%; T: 19.8 ± 0.5°C) following an overnight fast (8–14 h) during two consecutive mornings. Participants were instructed to lie supine on a bed for ~30 min in a quiet, darkened room and were asked to breathe normally through a Hans-Rudolph mouthpiece with nose clip during the last 18 min. Respiratory measurements were collected using the metabolic cart and the last 15 min of data were analyzed for resting oxygen consumption (VO_2_) and carbon dioxide production (VCO_2_). Respiratory exchange ratio (RER) was calculated as VCO_2_/VO_2_. Whole body resting fatty acid (FA) oxidation, carbohydrate (CHO) oxidation, and resting metabolic rate (RMR) were calculated using the following equations [21]:

$$FA\;oxidation\;\left(\frac{g}{min}\right)\;=\;\left[1.695\times VO2\;\left(\frac{L}{min}\right)\right]-\left[1.701\;\times VCO2\;\left(\frac{L}{min}\right)\right]$$

$$CHO\;oxidation\;\left(\frac{g}{min}\right)\;=\;\left[4.585\;\times VCO2\;(\frac{L}{min})\right]-\left[3.226\;\times VO2\;\left(\frac{L}{min}\right)\right]$$

$$RMR\;\left(kcal\right)\;=\;VO2\;\left(\frac{L}{min}\right)\times RER\;caloric\;equivalent\;\left(\frac{kcal}{L}\right)\times time\;\left(min\right)$$

### Blood analysis

Fasted serum samples were sent to LifeLabs Medical Laboratory Services (Kitchener, Ont., Canada) immediately after collection and analyzed for metabolic and inflammatory markers including triglycerides (TG), total cholesterol, low-density lipoprotein-cholesterol (LDL-c), high-density lipoprotein-cholesterol (HDL-c), the cholesterol/HDL-c ratio, glucose, and high sensitivity C-reactive protein (hsCRP).

For the analysis of fatty acids (FAs), a second aliquot of blood was centrifuged to separate plasma and red blood cells (RBCs). These fractions were aliquoted and stored at −80°C until further analysis. The FA profile of RBC samples was measured by gas chromatography as previously described [22]. FA peaks were identified by comparison to retention times of FA methyl ester standards. Fatty acids were expressed as percent of total FAs.

### Statistical analysis

A paired t-test was used for calculating sample size. To detect a clinically important difference of 26 ml/min of VO_2_, assuming a standard deviation of 24 ml/min [23], a power of 80% and a significance level of 5% (two-sided), a sample size of 12 participants per group was sufficient. The calculation was based on the assumption that the measurements on VO_2_ were normally distributed.

All data are presented as means ± standard deviation (SD). Intra-individual coefficient of variation (CVintra) from consecutive day measurements was calculated as [(SD/mean) x 100]. A one-way repeated measures ANOVA was performed to determine the effect of time on CVintra for both groups. When significance was found, Tukey post hoc tests were used.

Paired t-test comparisons were performed on OO and FO groups to determine if any differences existed between consecutive day measurements at weeks 0, 6 and 12, and for blood analyses at weeks 0 and 12. Unpaired t-tests were performed between summer and winter groups at baseline (Week 0) to determine if any differences existed on metabolic measurements.

A two-way repeated measures ANOVA with a post-hoc Tukey test was used to examine changes in metabolic measurements and RBC fatty acid profiles using treatment (OO—FO or summer—winter) and time (Weeks 0–6–12) as fixed effects.

Statistical significance was declared at the 0.05 level. GraphPad Prism program, version 6.0 (GraphPad Software, Inc., La Jolla, CA, USA) was used for statistical analysis. All data were checked for normality before any analysis was performed.

## Results

### Subject characteristics and blood analysis

The subjects from the OO and FO supplementation groups were well matched at week 0 for physical characteristics and blood measurements (Table 1). Supplementation for 12 weeks had no effect on these measures in either group, except for a reduced TG concentration (~20%, p = 0.04) in the FO group (Table 1).

### Fatty acid analysis of red blood cells

**Fig 2:**
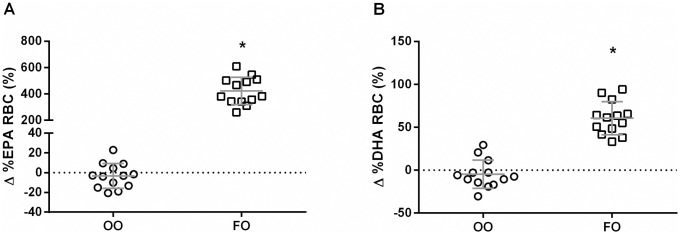
Percent (%) increase in red blood cell (RBC) levels of EPA (A) and DHA (B) in olive oil (OO) and fish oil (FO) groups after 12 weeks of supplementation. Values are reported as means ± SD.* FO group means were significantly different from the baseline (p < 0.001).

There were no group differences on RBC fatty acid composition of EPA (p = 0.99) and DHA (p = 0.99) at baseline. RBC levels of EPA were increased in the FO group from 0.6 (week 0) to 3.1% (week 12) of total FAs (p < 0.001). Similarly DHA levels in RBC were increased from 2.9 to 4.6% of total FAs (p < 0.001). There was no effect of OO supplementation on EPA (p = 0.99) and DHA (p = 0.86) RBC levels (Fig 2).

### Metabolic measurements

#### Day-to-day variability

There was a high degree of repeatability between consecutive day metabolic measurements at weeks 0, 6 and 12, as there were no differences between days (Tables 2 and 3). The one exception was FAOx in the FO group at week 0 which was higher (p = 0.02) on day 2 (Table 3).

**Table 2:** Resting metabolic rate, substrate oxidation, and within individual coefficient of variance between consecutive day measurements with olive oil supplementation.

Olive Oil																		
	Week 0 (n = 12)						Week 6 (n = 13)						Week 12 (n = 13)					
	Day 1		Day 2		CV intra (%)		Day 1		Day 2		CV intra (%)		Day 1		Day 2		CV intra (%)	
	Mean	SD	Mean	SD	Mean	SD	Mean	SD	Mean	SD	Mean	SD	Mean	SD	Mean	SD	Mean	SD
**VO**_**2**_	285	35	289	32	2.75	1.38	286	28	289	32	2.65	1.95	293	30	289	29	2.51	1.71
**VCO**_**2**_	240	33	246	27	3.17	1.51	246	30	248	31	3.76	2.56	246	24	243	25	2.41	2.02
**RER**	0.842	0.034	0.853	0.034	2.16	2.08	0.858	0.030	0.859	0.040	1.87	1.71	0.842	0.050	0.842	0.050	2.51	1.02
**RMR**	1990	250	2023	224	2.57	1.32	2006	204	2029	227	2.77	2.04	2044	201	2016	196	2.18	1.82
**CHO oxidation**	10.90	3.20	11.84	2.65	13.23	11.08	12.28	3.54	12.35	3.57	11.55	10.10	11.06	3.73	10.87	4.05	17.85	10.65
**FA oxidation**	4.47	0.98	4.24	1.14	13.36	13.59	3.99	0.95	4.06	1.21	12.80	10.02	4.65	1.67	4.60	1.78	15.65	7.01

VO_2_ (ml/min), resting oxygen consumption; VCO_2_ (ml/min), resting carbon dioxide production; RER, respiratory exchange ratio, RMR (kcal/day), resting metabolic rate; CHO oxidation (g/h), carbohydrate oxidation; FA oxidation (g/h), fatty acid oxidation.

All values are means ± SD.

**Table 3:** Resting metabolic rate, substrate oxidation, and within individual coefficient of variance between consecutive day measurements with fish oil supplementation.

Fish Oil																		
	Week 0 (n = 12)						Week 6 (n = 13)						Week 12 (n = 13)					
	Day 1		Day 2		CV intra (%)		Day 1		Day 2		CV intra (%)		Day 1		Day 2		CV intra (%)	
	Mean	SD	Mean	SD	Mean	SD	Mean	SD	Mean	SD	Mean	SD	Mean	SD	Mean	SD	Mean	SD
**VO**_**2**_	289	26	298	32	2.93	2.35	291	24	295	33	3.96	3.47	295	30	299	23	3.16	2.89
**VCO**_**2**_	243	23	244	28	2.44	3.19	245	22	247	26	4.11	3.20	239	26	243	17	4.89	3.69
**RER**	0.839	0.048	0.821	0.036	2.33	1.56	0.841	0.040	0.840	0.040	1.76	1.11	0.811	0.050	0.815	0.050	4.04	2.28
**RMR**	2019	176	2069	224	2.70	2.44	2033	165	2059	228	3.98	3.30	2044	203	2073	150	3.22	2.97
**CHO oxidation**	10.75	3.79	9.61	3.06	15.27	7.97	10.95	3.05	10.94	2.93	11.64	9.75	8.57	4.44	8.98	3.83	33.66∞	16.00
**FA oxidation**	4.66	1.57	5.34*	1.29	14.81	14.74	4.64	1.23	4.76	1.44	11.29	8.46	5.64	1.93	5.60	1.80	21.51	14.34

VO_2_ (ml/min), resting oxygen consumption; VCO_2_ (ml/min), resting carbon dioxide production; RER, respiratory exchange ratio, RMR (kcal/day), resting metabolic rate; CHO oxidation (g/h), carbohydrate oxidation; FA oxidation (g/h), fatty acid oxidation.

All values are means ± SD.

* Significant difference (p = 0.02) between day 1 and day 2.

^∞^ Significantly different from week 0 and week 6 (p < 0.001).

The mean intra-individual coefficient of variation (CVintra) ranged from 1.76–4.89% for VO_2_, VCO_2_, RER, and RMR after consecutive morning measurements on three different occasions. This variation was similar in the OO and FO groups (Tables 2 and 3). CVintra for FA oxidation ranged from 12.80–15.65% in the OO group and 11.29–21.51% in the FO group. The CVintra for CHO oxidation was similar and ranged from 11.55–17.85% in the OO group and 11.64–15.27% in the FO group, with the exception of week 12 (33.66%) which was greater (p < 0.001) than weeks 0 and 6 (Table 3).

#### Resting metabolic rate normalized to body mass

**Fig 3:**
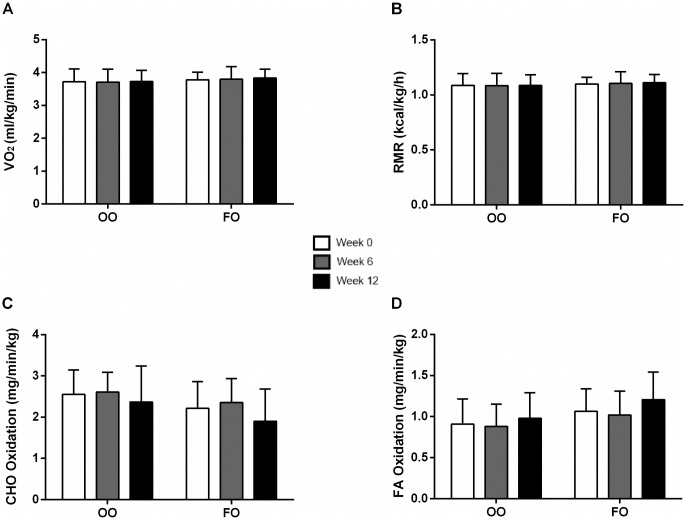
Resting oxygen consumption (VO_2_) (A), resting metabolic rate (RMR) (B), carbohydrate (CHO) oxidation (C), and fatty acid (FA) oxidation (D) normalized to body mass at weeks 0, 6 and 12 of olive oil (OO) and fish oil (FO) supplementation. Values are reported as means ± SD. There were no differences between the groups.

**Fig 4:**
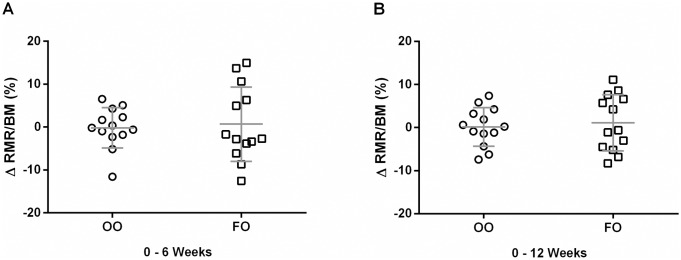
Change (%) in resting metabolic rate (RMR) normalized to body mass (BM) after 6 (A) and 12 (B) week supplementation of olive oil (OO) and fish oil (FO). Values are reported as means ± SD. There were no differences between the groups.

Since there was a high degree of repeatability between day to day measurements and there was no change in body mass (BM) during the 12 week supplementation period (Tables 1–3), the data were averaged and normalized to BM for each subject. The mean VO_2_, RMR, and substrate oxidation did not change following 6 and 12 weeks of FO or OO supplementation (Figs 3A, 3B, 3C, 3D, 4A and 4B).

#### Seasonal effects on RMR and substrate oxidation normalized to body mass

**Fig 5:**
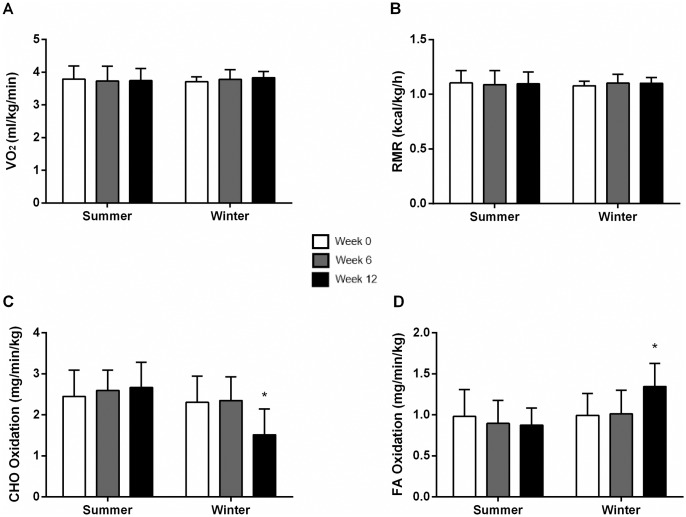
Resting oxygen consumption (VO2) (A), resting metabolic rate (RMR) (B), carbohydrate (CHO) oxidation (C), and fatty acid (FA) oxidation (D) normalized to body mass at weeks 0, 6 and 12 in summer and winter groups. Values are reported as means ± SD. *Significantly different from weeks 0 and 6 (Winter) and weeks 0, 6 and 12 (Summer) (p<0.01).

Since there was no effect of supplementation on metabolic measures with either OO or FO, we pooled the two treatment groups to determine whether there was a seasonal effect on RMR and substrate oxidation. A group of 14 subjects tested in the summer (n = 7 OO and n = 7 FO) and 12 tested in the winter (n = 6 OO and n = 6 FO) were analyzed. There was no difference between groups at baseline (p > 0.1). There was no change in VO_2_ or RMR after 6 and 12 weeks (Fig 5A and 5B) in the summer or winter, and also no change in substrate oxidation over the 12 weeks in the summer group. However, there was an increase in FA oxidation (36%) with a concomitant decrease in CHO oxidation (34%) at week 12 in the winter group when compared to weeks 0 and 6, and also when compared to the summer group (p < 0.01) (Fig 5C and 5D). There was no change in daily energy intake and diet composition before and after 12 weeks in both groups.

## Discussion

The primary finding of this well-controlled study was that 12 weeks of FO supplementation had no effect on RMR and substrate oxidation in young, healthy males. A secondary finding was that FA oxidation was increased and carbohydrate oxidation decreased over a 12 week period during the winter season, independent of supplementation and with no change in energy expenditure. In addition, this study determined that the intra-individual coefficient of variability (CVintra) between repeat measures was 2–4% for RMR and 11–22% for whole body substrate oxidation prior to and following 6 and 12 weeks of supplementation.

### Variability of metabolic measurements

One of the problems with studying whole body energy expenditure and substrate oxidation is the biological and measurement variability that exists between day to day measurements [24]. In fact, the potential magnitude of any omega-3 effects on RMR and substrate oxidation may be at or near the documented day-to-day variability [15,16,24–28]. In the present study, the CVintra for RMR on two consecutive mornings at 3 different time points was between 2–4%. These results are in agreement with previous studies where the CVintra after consecutive measurements (1–14 days apart) was between 1–5% in healthy humans [25–29]. The variability of whole body substrate oxidation was higher, fluctuating from 11–22%, similar to a previous study were macronutrient oxidation ranged from 15–25% [19]. Therefore, there was tight repeatability between day-to-day procedures, and to observe a meaningful effect of FO supplementation in healthy young males, RMR and substrate oxidation changes needed to be greater than 4% and 22%, respectively.

### Effect of fish oil supplementation on metabolic measurements

The previous work that examined the effects of FO supplementation in healthy young adults on whole body RMR and FA oxidation is limited and controversial. For example, Couet et al. [15] reported that 3 weeks of FO supplementation (1.1 g/d EPA and 0.7 g/d DHA) in healthy young adults (5 men, 1 woman) significantly increased RMR (4%) and FA oxidation (22%). However, when RMR was normalized to lean body mass, the effects of FO were no longer significant. In contrast, Bortolotti et al. [17] reported no change in RMR and FA oxidation after a 15 day FO supplementation period (1.1 g/d EPA, 0.7 g/d DHA) in healthy young males (n = 8). However, these measurements were done after feeding the participants a high carbohydrate meal prior to testing, which may have confounded the results. Noreen and coworkers [18] also found no changes in either RMR or substrate oxidation during a longer FO supplementation period (6 weeks; 1.6 g/d EPA, 0.8 g/d DHA) in both healthy adult males (n = 6) and females (n = 16). Conversely, a recent study by our group using a healthy young population (n = 21) revealed that FO supplementation (2 g/d EPA, 1 g/d DHA) for 12 weeks significantly increased RMR by 5%, with no effect on FA and CHO oxidation, and no changes in the olive oil control group (n = 9) [16]. However, when RMR was normalized to body mass, the 5% change was no longer statistically significant, similar to the findings of Couet et al. [15].

In the present study, in order to minimize biological and measurement variability, subjects were matched for age, body mass, height, and metabolic measurements (Table 1) and randomly assigned to the OO or FO group. Both groups also received high doses of FO or OO, continued the supplementation for longer (12 weeks) than most previous studies, and any potential seasonal effects were controlled for. All metabolic testing was also conducted with paired subjects, with one subject from the FO group and one from the OO group measured at the same time. Lastly, metabolic measurements were also done on two consecutive days at baseline and after 6 and 12 weeks of supplementation. With these methodological controls in place, we found no effect of FO supplementation on whole body RMR and substrate oxidation at either 6 or 12 weeks in young healthy males.

### Omega-3 supplementation and resting skeletal muscle metabolism

Although the content of omega-3s in skeletal muscle membranes was not measured, previous studies have demonstrated incorporation of omega-3s into whole muscle [30], as well as mitochondrial [31] and sarcolemmal [unpublished results] membranes after 4–12 weeks of FO supplementation. The incorporation into muscle was positively correlated with an increase in EPA and DHA in RBC membranes [32]. In the present study, after 12 weeks of supplementation, there was a significant increase of omega-3s in RBCs (Fig 2), making it likely that these FAs were also incorporated into skeletal muscle membranes. It has been hypothesized that a higher content of omega-3s in skeletal muscle may increase whole body energy expenditure by altering the activity of membrane bound proteins and/or increasing mitochondrial proton leak [8]. However, this study detected no change in whole body RMR after FO supplementation, suggesting that the incorporation of omega-3s into muscle membranes of healthy young males may have a small or null effect on protein activity and proton leak. For instance, previous studies suggested increased maximal Na/K-ATPase activity in membranes with a high DHA content [8] however, this does not reflect the in vivo situation that is present during basal energy expenditure, where the activity of this protein is very low [33]. In addition, recent work from our laboratory demonstrated that mitochondrial leak assessed with permeabilized muscle fibers was not changed after 12 weeks of omega-3 supplementation in healthy young males. This is consistent with other studies in both rodents [34] and insulin resistant humans [35].

Interestingly, we also recently reported that 6 and 12 weeks of FO supplementation significantly increased RMR in both older females [23] and males [unpublished results] (60–75 y), compared to olive oil control groups. It therefore seems possible that omega-3s may be incorporated to a greater degree in skeletal muscle of older individuals. Future research should determine whether incorporation of omega-3s into skeletal muscle in older adults decreases mitochondrial efficiency and increases the activity of membrane bound ATPases. However, it must be noted that other organs may also be affected by FO supplementation and contribute to the increase in RMR.

### Seasonal effects on resting metabolic measurements

Ambient temperature is known to influence RMR and substrate oxidation when measured in a thermoneutral environment. RMR also appears to be influenced by geographical location and variability between populations [36,37]. Plasqui et al. [38] reported that healthy, young Dutch participants had a significantly higher (6%) sleeping metabolic rate during winter compared to summer, while RER did not change between seasons. Similarly, Nishimura et al. [39] showed that healthy Japanese males increased their resting VO_2_, but had a significantly lower RER, during the winter season in comparison to summer, when measured in a thermoneutral environment. In the present study, there was no change in energy expenditure after 12 weeks in both groups, however there was a significant increase in FA oxidation (36%) with a concomitant decrease in CHO oxidation (34%) in the winter group, independent of supplementation. A possible explanation for the increase in FA oxidation may be due to the increasing cold exposure experienced by the participants during the months that bracketed this 12 week study—average monthly outdoor temperatures of +9.2, +0.1, -1.4 and -9.0°C. Indeed, during chronic cold exposure there is an increased β-adrenergic response [40] which may lead to increased lipolysis and whole body fatty acid oxidation [41]. Future research should focus on the possible mechanisms and tissues involved in this adaptive response.

The authors acknowledge that the present study has limitations. The use of olive oil as a placebo could have some biological effects, however a previous study from our laboratory used the same dose and observed no change in either whole body RMR or substrate oxidation [16]. Additionally, this study detected no significant change of RBC fatty acid content after olive oil supplementation. If olive oil has any metabolic effects, it is unlikely due to changes in membrane composition. Lifestyle factors such as diet, exercise, and stress can also impact whole body RMR. However, in this study these factors were minimized in an attempt to maximize the accuracy of metabolic measurements.

In conclusion, our results indicate that when biological and measurement variability was minimized, 12 weeks of FO supplementation did not affect RMR and substrate oxidation in healthy young males. There was a seasonal effect, independent of supplementation, on whole body substrate oxidation, where fatty acid oxidation increased and carbohydrate oxidation decreased over a 12 week period during winter, when measured in a thermoneutral environment.

## Supporting information

S1 CONSORT Checklist (DOC)

S1 Trial Protocol (DOCX)
