# Supplementary material for: Lack of effects of fish oil supplementation for 12 weeks on resting metabolic rate and substrate oxidation in healthy young men: A randomized controlled trial
Source: PLoS One. 2017 Feb 17;12(2):e0172576. doi: 10.1371/journal.pone.0172576 (PMC5315390; doi:10.1371/journal.pone.0172576)
Supplement: S1 Trial Protocol — (DOCX) [file pone.0172576.s002.docx]

University of Guelph

Research Ethics Board (REB)

Application to Involve Human Participants in Research (REBApp)

# **SECTION A: ADMINISTRATIVE INFORMATION** [
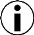
](http://www.uoguelph.ca/research/services-divisions/ethics/reb-app-guidelin#se)

A.1 Title of the research project: Effects of 12 weeks of Omega 3 Supplementation on Resting Metabolic Rate [
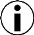
](http://www.uoguelph.ca/research/services-divisions/ethics/reb-app-guidelin#secaque)

A.2 Investigator Information [
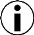
](http://www.uoguelph.ca/research/services-divisions/ethics/reb-app-guidelin#secaque)

| **Name & position** | **Principal Investigator** | **Faculty Co-Investigator** | **Student Investigator** | **Other Investigator** | **Department** | **Phone No.** | **E-Mail** | **Receives Communications** |
| --- | --- | --- | --- | --- | --- | --- | --- | --- |
| Lawrence Spriet  Chair | Yes |  |  |  | HHNS | x 53745 | lspriet@uoguelph.ca | Yes |
| Sebastian Jannas Vela | n/a |  |  |  | HHNS | x 53907 | sjannas@uoguelph.ca | Yes |
| Greg Eskedjian | n/a |  |  |  | HHNS | x 53907 | geskedji@uoguelph.ca | Yes |
| Stephanie Boville | n/a |  |  |  | HHNS | x53907 | sboville@uoguelph.ca | Yes |
| Dr. David Mutch | n/a |  |  |  | HHNS | x 53322 | dmutch@uoguelph.ca | Yes |
| Kaitlin Roke | n/a |  |  |  | HHNS | x 58015 | kroke@uoguelph.ca | Yes |
|  | n/a |  |  |  |  |  |  | Yes |
|  | n/a |  |  |  |  |  |  | Yes |

Provide name of person who completed this form: Sebastian Jannas Vela

A.3 Are there any issues or concerns regarding the timeline for approval that you would like to raise? N/A [
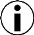
](http://www.uoguelph.ca/research/services-divisions/ethics/reb-app-guidelin#secaque)

|  |
| --- |

##### A.4 Research Ethics Approval (other than University of Guelph)

A.4.1 Will any other Research Ethics Board be asked for approval?  Yes  No [
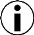
](http://www.uoguelph.ca/research/services-divisions/ethics/reb-app-guidelin#secaque0)

If **YES**, please specify:

|  |
| --- |

Copy of the clearance certificate or approval will be provided to the REB when available

Yes

Attached

A.4.2 If you are undertaking research in a country other than Canada, submit a copy of the clearance certificate/approval from the Research Ethics Board in that country. [
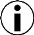
](http://www.uoguelph.ca/research/services-divisions/ethics/reb-app-guidelin#secaque0)

Attached

**OR** discuss what alternative measures are being taken (see information guide):

|  |
| --- |

## **A.5 Level of the Project:** please check all that apply [
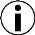
](http://www.uoguelph.ca/research/services-divisions/ethics/reb-app-guidelin#secaque)

|  | Faculty Research |
| --- | --- |
|  | PhD Thesis |
|  | Master’s Thesis |
|  | Masters Major Research Paper |
|  | M.Sc by Coursework |
|  | Honours Thesis |
|  | Class Project Specify course: |
|  | Internship |
|  | Practicum |
|  | Independent Study |
|  | Administration |
|  | Contract – for profit sponsor |
|  | Other – please specify: |

## **A.6 Funding of Project** [
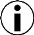
](http://www.uoguelph.ca/research/services-divisions/ethics/reb-app-guidelin#secaque)

A.6.1 Has funding been granted for this project?   Yes  No

Pending

A.6.2 Agency or Sponsor

| **Funder** | **Name of Program** | **Title of Grant** | **Grant Number** |
| --- | --- | --- | --- |
| NSERC | Discovery Grant | Exercise and nutritional control of lipid and mitochondrial metabolism in human skeletal muscle. |  |
|  |  |  |  |
|  |  |  |  |
|  |  |  |  |
|  |  |  |  |

Comments:/funder

|  |
| --- |

A.6.3 Contract – will there be an agreement with a research partner/funder (i.e. data sharing agreements, research funding agreements, confidentiality agreements etc.)? [
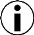
](http://www.uoguelph.ca/research/services-divisions/ethics/reb-app-guidelin#secaque0)

N/A

| Name of Research Partner/Sponsor: |
| --- |
| Title of Research Project: |
| Has a copy of the contract been submitted to the Contracts Department of the Office of Research?   Yes |
| Has the contract received final signatures?   Yes |
| Comments: |

## **A.7 Peer Review** [
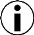
](http://www.uoguelph.ca/research/services-divisions/ethics/reb-app-guidelin#secaque)

| A.7.1 Has this project undergone peer review for scholarly merit during the course of funding approval? | Yes | No |
| --- | --- | --- |
| A.7.2 Has this project undergone peer review for scholarly merit by a graduate advisory committee? | Yes | No |
| A.7.3 Comments: | | |

## **A.8 Disclosure of Conflict of Interest** [
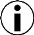
](http://www.uoguelph.ca/research/services-divisions/ethics/reb-app-guidelin#secaque)

A.8.1: Will the researcher(s), members of the research team, and/or their partners or immediate family members receive any personal benefits This might include a financial benefit such as remuneration/income, intellectual property rights, rights of employment, consultancies, board membership, share ownership, stock options etc.  Yes  No [
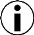
](http://www.uoguelph.ca/research/services-divisions/ethics/reb-app-guidelin#secaque0)

If YES, please describe the benefits below. Include details of all fees and/or honoraria directly related to this study, such as those for participant recruitment, advice on study design, presentation of results, or conference expenses.

|  |
| --- |

A.8.2 Describe any restrictions regarding access to or disclosure of information (during or at the end of the study) placed on the investigator(s), including those related to the publication of results. Note the nature of these restrictions and who is applying these restrictions.  N/A [
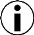
](http://www.uoguelph.ca/research/services-divisions/ethics/reb-app-guidelin#secaque0)

|  |
| --- |

A.8.3 Describe the possibility of commercialization of the research findings.  N/A [
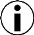
](http://www.uoguelph.ca/research/services-divisions/ethics/reb-app-guidelin#secaque0)

|  |
| --- |

A.8.4 Describe any personal or professional relationship between a member of the research team and any participants aside from the researcher/participant relationship. N/A [
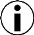
](http://www.uoguelph.ca/research/services-divisions/ethics/reb-app-guidelin#secaque0)

|  |
| --- |

A.8.5 Disclose any employment that research team members have outside the University of Guelph, if it is related in any way to the study (e.g. as the source of research participants.)  N/A [
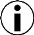
](http://www.uoguelph.ca/research/services-divisions/ethics/reb-app-guidelin#secaque0)

|  |
| --- |

A.8.6 Describe any consultancy or other contractual agreements, financial, partnership, or business interests within the last two years that might be perceived as a conflict of interest pertaining to this study. N/A [
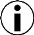
](http://www.uoguelph.ca/research/services-divisions/ethics/reb-app-guidelin#secaque0)

|  |
| --- |

A.8.7 Describe any patent rights or intellectual property rights linked in any way to this study or its sponsor that are held by investigators and/or their partners/immediate family members. N/A [
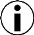
](http://www.uoguelph.ca/research/services-divisions/ethics/reb-app-guidelin#secaque0)

|  |
| --- |

## **A.9 Experience and Licensed** Qualifications **[
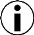
](http://www.uoguelph.ca/research/services-divisions/ethics/reb-app-guidelin#secaque)**

A.9.1 What experience does the principal investigator have with the kind of research undertaken in this project and in this context, including the nature of the participants, methods of data collection, etc.? [
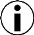
](http://www.uoguelph.ca/research/services-divisions/ethics/reb-app-guidelin#secaque0)

| The principal investigator (Dr. Spriet) has extensive experience with all of the measures listed in this study and the inherent risks. The student investigator (Sebastian Jannas Vela) and the other investigators are familiar with the questionnaires and physical function measures. Two similar protocols involving omega 3 supplementation and respiratory measures have been completed in the past 2 years (REB Numbers: 11SE032,12OC014). |
| --- |

A.9.2 What is the role of each member of the research team? [
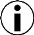
](http://www.uoguelph.ca/research/services-divisions/ethics/reb-app-guidelin#secaque0)

| **Name** | **Role** | **Contact with Identified Data** | **Direct Participant Contact** | **CORE Tutorial Completed** |
| --- | --- | --- | --- | --- |
| Lawrence Spriet | Principal Invesitgator - helper with study | Yes | Yes | Yes |
| Sebastian Jannas Vela | PhD Research - conducting the study | Yes | Yes | Yes |
| Greg Eskedjian | MSc student - helper with study | Yes | Yes | Yes |
| Stephanie Boville  David Mutch  Kaitlin Roke | MSc study - helper with study  Faculty co-investigator  PhD student - helper with the study | Yes | Yes | Yes |

A.9.3 How will the faculty with principal responsibility ensure that each team member has the expertise and experience necessary to carry out the research? How will s/he ensure that all team members are familiar with the contents of the ethics protocol? Discuss for each team member. [
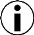
](http://www.uoguelph.ca/research/services-divisions/ethics/reb-app-guidelin#secaque0)

| **Name** | **Review confidentiality requirements** | **Review recruitment process** | **Review consent process** | **Direct oversight of procedure/process** | **Review of debrief post deception** | **Completion of graduate level method’s course** | **Other** | **Details:** |
| --- | --- | --- | --- | --- | --- | --- | --- | --- |
| Sebastian Jannas |  |  |  |  |  |  |  |  |
| Greg Eskedjian |  |  |  |  |  |  |  |  |
| Stephanie Boville |  |  |  |  |  |  |  |  |
| Kaitlin Roke |  |  |  |  |  |  |  |  |

If more space is required, please add information here:

|  |
| --- |

A.9.4 Does any specific procedure require professional expertise/recognized qualifications (e.g. performance of a controlled act)?  Yes  No

If **YES**, describe, and specify which team members have this expertise:

| Blood samples will be taken by trained phlebotomists: Premila Sathasivam - James Turgeon - Jamie-Lee Munroe. |
| --- |

# **SECTION B: SUMMARY**

Provide a summary below, of the research to be undertaken. Please do not attach copies of detailed proposals submitted to a funding agency or sponsoring agency protocols; these will not be reviewed.

B.1 Describe the purpose and background rationale for the proposed project, as well as any hypotheses and/or research question to be examined.

| Fatty acids are the main components of the cell membranes. It has been demonstrated that diet can alter the characteristics and function of many membranes in the body, which has an effect on cardiovascular and metabolic health. Over the last two decades there has been a substantial rise in the research of the effects of Omega 3 polyunsaturated fatty acids on bodily function. There are two fatty acids that are of particular interest to researchers, eicosapentanoic acid (EPA) and docosahexanoic acid (DHA). These fatty acids have unique unsaturated structures, and their incorporation into biological membranes seems to generate important and positive physiological effects. The body is unable to synthesize these fatty acids in high concentrations to elicit significant effects, so they must be obtained through diet in food or via supplementation.  It has been suggested that EPA and DHA supplementation increases resting metabolic rate (RMR) in humans, in part by increasing the use of fat as a fuel during rest. There are a limited number of studies examining the effects of Omega 3 supplementation on RMR. Some have found an increase in RMR while others have found no change. These studies have some limitations, as that they have either used a small sample size, a low omega fatty acid dose and/or short supplementation periods.  Recently our laboratory revealed that 12 weeks of Omega 3 supplementation (3g/d) had no effect on group mean RMR in healthy young men. However, there were increases in some of the subjects, suggesting a variable effect among healthy young men. A second study done in our lab showed a significant increase in RMR and fat oxidation in older adults at 6 and 12 weeks of supplementation (3g/d). Interestingly in healthy young adults, both the omega and placebo group increased their RMR. One of the limitations of this study was that it was done between two semesters (fall and winter). There are major lifestyle and climate changes that occur during these two periods, this might have had an effect on the RMR measurements. Due to the variable results, we will improve the reliability of the RMR measurements by making measures on each subject during two consecutive days at each time point that it is measured (0, 6 and 12 weeks).  Genetic variation in specific genes associated with fatty acid metabolism (i.e. the FADS1/2 gene cluster) will be examined using real time PCR. In addition, gene expression profiles will be analyzed from peripheral blood mononuclear cells isolated from fasted blood samples at baseline and at the end of the 3-month protocol. Briefly, blood will be drawn by an HNRU phlebotomist into a specialized vacutainer tube that contains a DNA stabilizer or an RNA stabilizer (these specialized vacutainer tubes and extraction kit are commercially available from Qiagen). These blood samples will be obtained at the same time and using the same venous sampling line as for the collection of other blood samples to measure blood lipids. Note that we only require this “DNA” blood sample to be obtained at a single time during the procedure (there is no evidence suggesting that DNA sequence changes with fish oil supplementation). As such, there is no additional risk to study participants. DNA and RNA will be quantified using NanoDrop 2000C apparatus and stored at -80°C until analysis (performed at the University of Guelph).  The methodologies used for the isolation of RNA and DNA from blood samples have been approved previously by the University of Guelph REB (protocol #11SE032 and #12JL006, respectively).  Therefore, the purpose of this study is to determine the effects of 12 weeks of omega 3 supplementation (3 g/d) on healthy young adults vs. the supplementation of a placebo. Please see attached product information for both capsules. The investigational products in use are manufactured, handled, and stored in accordance with applicable good manufacturing practice (GMP), and will be used in accordance with the approved protocol [Section 10]  We hypothesize that EPA and DHA supplementation will result in an increase in RMR and fat oxidation in some subjects and not in others. The duplicate RMR measures will determine the prevalence and magnitude of the omega 3 supplementation.  The Fish Oil Monograph (attached document) by Health Canada recommends a daily dose of a maximum of 5.000 mg for eicosapentaenoic acid (EPA) + docosahexaenoic acid (DHA). This project will supply a lower dose.  Furthermore, the supplementation rates described in the REBapp were approved by the Ethics Committee for two studies REB Numbers: 11SE032, 12OC014. This is a follow up study using same dosages and time of supplementation.  References  Bortolotti, M., Tappy, L., Schneiter, P., 2007. Fish oil supplementation does not alter energy efficiency in healthy males. Clinical nutrition 26, 225–30.  Gerling, C.J., Whitfield, J., Mukai, K., Chabowski, A., Heigenhauser, G.J.F, Holloway, G.P., and Spriet, L.L. 2014. Alterations in human skeletal muscle sarcolemmal and mitochondrial membrane fatty acid and phospholipid composition following omega-3 supplementation. Submitted, J Lipid Res.  Herbst, E.A., Paglialunga, S., Gerling, C., Whitfield, J., Mukai, K., Chabowski A., Heigenhauser, G.J., Spriet, L.L., and Holloway, G.P. 2014. Omega-3 supplementation alters mitochondrial membrane composition and respiration kinetics in human skeletal muscle. J Physiol. [Epub ahead of print].  Noreen, E.E., Sass, M.J., Crowe, M.L., Pabon, V.A., Brandauer, J., Averill, L.K., 2010. Effects of supplemental fish oil on resting metabolic rate, body composition, and salivary cortisol in healthy adults. Journal of the International Society of Sports. Nutrition 7, 31.  Raastad, T., Høstmark, A.T., Strømme, S.B., 1997. Omega-3 fatty acid supplementation does not improve maximal aerobic power, anaerobic threshold and running performance in well-trained soccer players. Scandinavian journal of medicine & science in sports 7, 25–31.  Surette, M.E., 2008. The science behind dietary omega-3 fatty acids. CMAJ : Canadian Medical Association journal 178, 177–80.  Zulyniak MA, Perreault M, Gerling C, Spriet LL, Mutch DM (2013) Fish oil supplementation alters circulating eicosanoid concentrations in young healthy men. Metabolism. Aug; 62(8): 1107-13. |
| --- |

B.2 Describe in clear and concise detail and sequentially each of the procedures in which the research participants will be involved. Use flow charts, diagrams, and/or point form. [
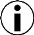
](http://www.uoguelph.ca/research/services-divisions/ethics/reb-app-guidelin#secbque)

| Procedure 1  - Participants will respond to posters by contacting the Student Investigator of the research team.  During the preliminary visit the researcher will explain the study protocol and requirements to the potential participants.  Subjects must complete 3-day dietary record; a participant screening form and an omega-3 questionnaire (documents attached) in order to determine eligibility to participate. Subjects will then visit the laboratory twice to complete a practice RMR measurement and a maximum oxygen consumption test (VO2 max). They will hand in their questionnaire and 3-day dietary record at this time. At this time, subjects will be asked to complete the genomic consent form (attached).  Subjects will be matched by age, body mass index (BMI), RMR, and VO2 max. Then they will be randomly assigned in a single-blinded manner to one of two supplement groups: Fish oil (omega 3) and Placebo (olive oil). The fish oil group will take 5 g/day of fish oil administered in 5 capsules, with each capsule providing 400 mg of EPA and 200 mg of DHA (3 g/day). The placebo group will take 3 g/d of olive oil administered in 3 capsules. |
| --- |

| Procedure 2 - Visits 1 and 2 (24 hours apart)  The subjects will arrive to the laboratory, between 7-11 am following an overnight fast (~10-12 hours). They will be instructed to lay supine on a bed in a darkened room for ~30 min, providing breath samples during the last 15 min. Heart rate will be measured every 5 min. Prior to these measurements, body mass and height of each participant will be determined. After the initial 30 min of lying on the bed, a blood sample (approximately 20 mL) will also be taken by a phlebotomist in the HNRU. |
| --- |

| Procedure 3 - Omegas and placebo - Visits 3 and 4 (24 hours apart) following 6 weeks of supplementation  Same protocol as Procedure 2. |
| --- |

| Procedure 4 Visit 5 and 6 (24 hours apart) following 12 weeks of supplementation  Same protocol as Procedure 2.  Subjects must come for an additional visit to perform a VO2 max test, no later than one week from cessation of supplementation. |
| --- |

| Procedure 5 Visit 7 Subjects must come for an additional visit to perform a VO2 max test, no later than one week after cessation of supplementation. |
| --- |

B.3 Indicate the location(s) where the research will be conducted (*check all that apply*): [
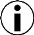
](http://www.uoguelph.ca/research/services-divisions/ethics/reb-app-guidelin#secbque)

|  | University of Guelph |
| --- | --- |
|  | South Western Ontario |
|  | Ontario |
|  | Canada |
|  | State Country: |

|  | Participant’s home |
| --- | --- |
|  | Participant’s place of business or workplace |
|  | School |
|  | University or College |
|  | Health Institution |
|  | Correctional Institution |
|  | Senior’s Institution |
|  | Other – please describe: Human Nutraceutical Research Unit |

SECTION C: METHOD [
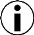
](http://www.uoguelph.ca/research/services-divisions/ethics/reb-app-guidelin#se)

Answer each question below for each of the procedures/methods discussed in Part B.

C.1 Time required of participants  N/A [
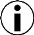
](http://www.uoguelph.ca/research/services-divisions/ethics/reb-app-guidelin#seccque)

For each type of interaction listed in B.2, describe the time required of participants. Also state the

total time required over all interactions:

| Procedure 1: 2,5 hours  Procedure 2: 2.5 hours  Procedure 3: 2.5 hours  Procedure 4: 2.0 hours  Procedure 5: 1.0 hours  Total Hours: 12 |
| --- |

C.1.1 Do you plan to re-contact participants for any purpose? If **YES**, this must be discussed here and in the consent form.

| No |
| --- |

## C.2 Language [
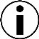
](http://www.uoguelph.ca/research/services-divisions/ethics/reb-app-guidelin#seccque)

In what language(s) will the research be conducted?  English

French

Other

N/A

C.2.1 Is the participant sufficiently fluent in this language to understand the consent process?  Yes  No

C.2.2 If not, is interpretation available?   Yes  No

C.2.2.1How will interpreter(s) be recruited? From what organization? From what region and cultural background? [
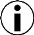
](http://www.uoguelph.ca/research/services-divisions/ethics/reb-app-guidelin#seccque02)

|  |
| --- |

C.2.2.2 Discuss the possible relationship between the interpreter(s) and the participants.

|  |
| --- |

C.2.2.3 Sample of Confidentiality Agreement or script for interpreter is: [
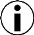
](http://www.uoguelph.ca/research/services-divisions/ethics/reb-app-guidelin#seccque02)

attached

pending – will be provided to the REB.

C.2.2.4 Project documents (such as consent forms, information letters, surveys) should, where possible, should be made available to participants in translation. Will this occur for this project?  Yes  No [
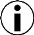
](http://www.uoguelph.ca/research/services-divisions/ethics/reb-app-guidelin#seccque02)

If NO, explain:

| The study will be done on male university students that are studying in the University of Guelph and/or volunteers from the general Guelph community and therefore translation will not be necessary. |
| --- |

## **C.3 Participants** [
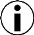
](http://www.uoguelph.ca/research/services-divisions/ethics/reb-app-guidelin#seccque)

C.3.1 Estimate the number of participants you will be recruiting 24

C.3.2 Estimate the size of the pool from which you are drawing participants, if possible

C.3.3 Will you be recruiting either males only or females only?  Yes  No  N/A [
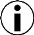
](http://www.uoguelph.ca/research/services-divisions/ethics/reb-app-guidelin#seccque0)

If **YES**, please state the rationale:

| Females will not be included in this study, but a follow-up study is planned with young female subjects. This study follows on a first study with males that was inconclusive. |
| --- |

C.3.4 What is the age range of the participants you will be recruiting?  N/A

Lower Age Limit: 18

Upper Age Limit: 30

Justify both the upper and lower limit. Children and the elderly should not automatically be excluded from research based solely upon their age.

| We are expanding previous work and findings done on our lab, where the subjects that participated were between 18 - 30 years old. We chose consenting young adults, therefore children under 18 years old will be exluded. |
| --- |

C.3.5 Are participants affiliated with (formally or informally) a particular organization/institution?  Yes  No

If **YES**, please name and provide details of the affiliation:

| Students of University of Guelph - but others will simply be from the City of Guelph community |
| --- |

C.3.6 Participant Inclusion and Exclusion Criteria: List all inclusion/exclusion criteria. [
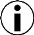
](http://www.uoguelph.ca/research/services-divisions/ethics/reb-app-guidelin#seccque0)Indicate with an asterisk (*) those criteria which will be included in the Letter of Information.

| **Inclusion Criteria** | **Exclusion Criteria** |
| --- | --- |
| History/General Medical Health *  Have no medical condition, hospitalization, allergy to fish oil, or surgeries. | Taken any medications, have any medical condition, and hospitalization or surgeries*  Allergy to fish/fish oil, , (rosemary extract, ascorbyl palmitate, or natural tocopherols)* |
| Age*  Between 18 and 30 years old | Under 18 or over 30 years old. |
| Omega 3, diet or supplementation*  Consume less than two meals of fish/wk and/or have not taken an omega-3 supplement during the prior three months* | Consume more than two meals of fish/wk and/or have taken an omega-3 supplement during the prior three months* |

# **C.4 Recruitment**

C.4.1 What form will recruitment take: (*please check all that apply)*:  N/A [
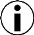
](http://www.uoguelph.ca/research/services-divisions/ethics/reb-app-guidelin#seccque0)

|  | Poster |
| --- | --- |
|  | Advertisement |
|  | Email |
|  | Web page |
|  | Letter of Invitation |
|  | Telephone Call |
|  | Social Media |
|  | Verbal Script |
|  | [Office of Research Participant Recruitment Site](http://www.uoguelph.ca/research/Research-Participants-Needed) |
|  | Other – describe below |

Attach a copy of the above with your submission.

Describe how/where you will use each of the instruments selected above:

| Subjects will be recruited with posters placed in public areas around campus, such as within the Department of Human Health and Nutritional Science, the Athletic Center, and University Center and in locations around the City of Guelph. |
| --- |

C.4.2 Indicate the location of the participant at the time of recruitment. Is the physical location of the participant at the time of recruitment of importance? For example, could contacting the individual at their place of business increase risk of harm? N/A [
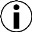
](http://www.uoguelph.ca/research/services-divisions/ethics/reb-app-guidelin#seccque0)

|  | At home |
| --- | --- |
|  | At work |
|  | Other – describe below |

Discuss for each of the instruments selected in C.4.1, as appropriate:

|  |
| --- |

C.4.3 If you have used Mass Testing as a recruitment tool, discuss the selection criteria used, and provide the REB number under which the mass testing item was approved.

N/A

|  |
| --- |

C.5 Incentives and Reimbursement [
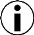
](http://www.uoguelph.ca/research/services-divisions/ethics/reb-app-guidelin#seccque)

C.5.1 What is the dollar value of incentive payments and other forms of reimbursement to participants? [
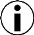
](http://www.uoguelph.ca/research/services-divisions/ethics/reb-app-guidelin#seccque0)

|  | Participants will be reimbursed for costs incurred while participating |
| --- | --- |
|  | Travel:  Child Care:  Parking:  Other: |
|  | Participants will receive incentives to encourage participation |
|  | Gift card:  Cash: Participants will receive compensation after the third procedure and after the fourth procedure. 50 dollars per procedure (Total: 100 dollars) Lottery or draw:       If yes, describe in C.5.2. Other: |
|  | Participants will receive non-financial benefits |
|  | Food and Drink: They will receive a cereal bar and juice after each procedural visit.  Other: Feedback of their RMR status. |
|  | Other – describe: |

C.5.2 If you have indicated in C.5.1 that you will be using a Lottery or Draw, please [
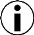
](http://www.uoguelph.ca/research/services-divisions/ethics/reb-app-guidelin#seccque0)provide the following information:

Estimated chances of winning

Number of prizes

Value of prizes

Give detailed description of how draw will be managed.

|  |
| --- |

C.5.3 If you have indicated in C.5.1 that you will be providing payment to participants, how will you record dispersal of funds for audit purposes (i.e. reporting to Financial Services)?

[
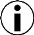
](http://www.uoguelph.ca/research/services-divisions/ethics/reb-app-guidelin#seccque0)

| Participants will be paid in the form of cheques prepared by Financial Services from a NSERC trust fund. |
| --- |

C.5.4 If you have indicated any incentives or reimbursement in C.5.1, detail how will you deal with incentives, reimbursements if participants choose to withdraw? (Cash payments should be prorated.) [
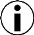
](http://www.uoguelph.ca/research/services-divisions/ethics/reb-app-guidelin#seccque0)

| Subjects will receive compensation of ~ 12.5 dollars per visit. Payment will be given after week 6 and week 12, 50 dollars per procedure (Total: 100 dollars). |
| --- |

C.5.5 Are the participants likely to incur any expenses or inconveniences in addition to those described above as a result of their participation in this project?  Yes  No [
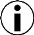
](http://www.uoguelph.ca/research/services-divisions/ethics/reb-app-guidelin#seccque0)

If **YES**, describe:

|  |
| --- |

SECTION D: THE INFORMED CONSENT PROCESS [
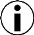
](http://www.uoguelph.ca/research/services-divisions/ethics/reb-app-guidelin#se)

Are you planning on providing participants with a hard copy consent document, which they will sign? If so, fill out D.2.

Are you planning on obtaining oral consent? If so, fill out D.3.

Are you planning a survey, which will display the consent information at the front of the survey, and you will assume participants consent if they complete and return the survey? If so, please fill out D.4.

Will your participants be unable to give consent themselves, but must have a parent or guardian give consent on their behalf? These participants might be children, or an adult with a cognitive impairment, for example. If this is the case, fill out D.5.

Section D.6 should only be filled out if your project involves deception. Please see [Guideline 1-G-020](http://www.uoguelph.ca/research/assets/ethics/documents/guidelines/1-G-020.pdf) for information about deception.

Section D.1 is seldom used, and is a waiver of prior informed consent. See the information guide for an explanation of when D.1 applies.

You may fill out more than one type of consent section. You need not fill out ALL consent sections – only what you need.

D.1.Alteration of Informed Consent: [
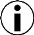
](http://www.uoguelph.ca/research/services-divisions/ethics/reb-app-guidelin#secdque)

If you are applying for a waiver of prior informed consent, see the information guide, and discuss Article 3.7 (a) to (f).  N/A

|  |
| --- |

D.2 Written Consent: Will you be obtaining consent with a signature?  Yes  No [
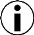
](http://www.uoguelph.ca/research/services-divisions/ethics/reb-app-guidelin#secdque)

If **NO**, please explain why signed consent is not appropriate in this case then go to D.3.

|  |
| --- |

If **YES** to consent with a signature, please answer the following:

D.2.1 What consent documents will be used to inform potential participants about the details of the project and to obtain consent for participation? [
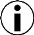
](http://www.uoguelph.ca/research/services-divisions/ethics/reb-app-guidelin#secdque0)

|  | Information letter, and consent form with signature section |
| --- | --- |
|  | Consent form with signature section |
|  | Other – Specify |

Discuss:

|  |
| --- |

D.2.2 How will consent documents be delivered to participant? [
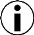
](http://www.uoguelph.ca/research/services-divisions/ethics/reb-app-guidelin#secdque0)

| They will be provided to the participant during the first visit (procedure). |
| --- |

D.2.3 How will consent documents be returned to researcher? [
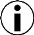
](http://www.uoguelph.ca/research/services-divisions/ethics/reb-app-guidelin#secdque0)

| During the first procedure, or at the end of the first meeting. |
| --- |

D.2.4 Which member of the research team will manage the consent process? [
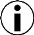
](http://www.uoguelph.ca/research/services-divisions/ethics/reb-app-guidelin#secdque0)

| Student Investigator. |
| --- |

D.2.5 Has this individual had the necessary training to administer consent? Describe the training received or planned. [
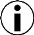
](http://www.uoguelph.ca/research/services-divisions/ethics/reb-app-guidelin#secdque0)

| Yes. |
| --- |

D.2.6 Verify the following: [
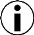
](http://www.uoguelph.ca/research/services-divisions/ethics/reb-app-guidelin#secdque0)

| Yes | N/A | Copy of consent form is attached to this application | Comment |
| --- | --- | --- | --- |
| Yes | N/A | Copy of consent form will be given  to participant | Comment Same as above |
| Yes | N/A | Script for introducing consent process is attached to this application | Comment |
| Yes | N/A | Letter of information is attached to this application | Comment |
| Yes | N/A | Copy of information letter shows University of Guelph letterhead or logo | Comment |

D.2.7 Will the participant have an opportunity to have questions about the project and their role as a participant answered? How will this opportunity be communicated to them?

N/A [
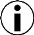
](http://www.uoguelph.ca/research/services-divisions/ethics/reb-app-guidelin#secdque0)

| Yes, during the first visit all our procedures will be explained and participants may ask any questions about the study. |
| --- |

If this written consent is from a parent or guardian, please fill out section D.5 as well.

D.3 Oral Consent: Will you be obtaining oral consent?  Yes  No [
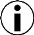
](http://www.uoguelph.ca/research/services-divisions/ethics/reb-app-guidelin#secdque)

If **NO**, please go to D.4

If **YES**, to oral consent please answer the following**:**

D.3.1 What documents will be used to provide participants with information about the project to supplement the oral consent? [
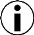
](http://www.uoguelph.ca/research/services-divisions/ethics/reb-app-guidelin#secdque0)

|  | Information letter |
| --- | --- |
|  | Consent script |
|  | Other – Specify |

Discuss:

|  |
| --- |

D.3.2 How will the written information be delivered to participant? [
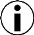
](http://www.uoguelph.ca/research/services-divisions/ethics/reb-app-guidelin#secdque0)

|  |
| --- |

D.3.3 How will oral consent be documented by the researcher? [
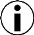
](http://www.uoguelph.ca/research/services-divisions/ethics/reb-app-guidelin#secdque0)

|  |
| --- |

D.3.4 Which member of the research team will administer consent? [
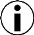
](http://www.uoguelph.ca/research/services-divisions/ethics/reb-app-guidelin#secdque0)

|  |
| --- |

D.3.5 Has this individual had the necessary training to administer consent? Describe the training received or planned. [
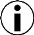
](http://www.uoguelph.ca/research/services-divisions/ethics/reb-app-guidelin#secdque0)

|  |
| --- |

D.3.6 Verify the following: [
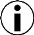
](http://www.uoguelph.ca/research/services-divisions/ethics/reb-app-guidelin#secdque0)

| Yes | N/A | Copy of consent script is attached to this application | Comment |
| --- | --- | --- | --- |
| Yes | N/A | Copy of information letter will be given to participant | Comment |
| Yes | N/A | Copy of information letter shows University of Guelph letterhead or logo | Comment |
| Yes | N/A | Copy of information letter is attached to this application | Comment |

D.3.7 Will the participant have an opportunity to have questions about the project and their role as a participant answered? How will this opportunity be communicated to them?

N/A [
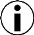
](http://www.uoguelph.ca/research/services-divisions/ethics/reb-app-guidelin#secdque0)

|  |
| --- |

D.4 Assumed Consent: Will consent be assumed or implied? Yes  No [
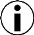
](http://www.uoguelph.ca/research/services-divisions/ethics/reb-app-guidelin#secdque)

If **NO**, please go to D.5

If **YES** to assumed or implied consent, please answer the following:

D.4.1 What consent documents will be used to provide potential participants written [
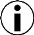
](http://www.uoguelph.ca/research/services-divisions/ethics/reb-app-guidelin#secdque0)information about the details of the project to supplement the assumed or implied consent?

|  | Information letter |
| --- | --- |
|  | Consent form |
|  | Other – Specify |

Discuss:

|  |
| --- |

D.4.2 How will the written information be delivered to participant? For online surveys, invite the participant to print the consent information. [
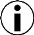
](http://www.uoguelph.ca/research/services-divisions/ethics/reb-app-guidelin#secdque0)

|  |
| --- |

D.4.3 How will consent be documented by the researcher (for example, by return of the completed questionnaire)? [
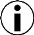
](http://www.uoguelph.ca/research/services-divisions/ethics/reb-app-guidelin#secdque0)

|  |
| --- |

D.4.4 Which member of the research team will administer consent?  N/A [
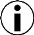
](http://www.uoguelph.ca/research/services-divisions/ethics/reb-app-guidelin#secdque0)

|  |
| --- |

D.4.5 Has this individual had the necessary training to administer consent? Describe the training received or planned.  Yes  No  N/A [
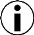
](http://www.uoguelph.ca/research/services-divisions/ethics/reb-app-guidelin#secdque0)

|  |
| --- |

D.4.6 Verify the following: [
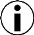
](http://www.uoguelph.ca/research/services-divisions/ethics/reb-app-guidelin#secdque0)

| Yes | N/A | Copy of information letter will be available to participant (provide PRINT button for online survey) | Comment |
| --- | --- | --- | --- |
| Yes | N/A | Copy of information letter shows University of Guelph letterhead or logo | Comment |

D.4.7 Will the participant have an opportunity to have questions about the project and their role as a participant answered? How will this opportunity be communicated to them?

N/A [
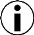
](http://www.uoguelph.ca/research/services-divisions/ethics/reb-app-guidelin#secdque0)

|  |
| --- |

D.5 Proxy Consent: Will you be obtaining proxy consent (e.g. of parent/guardian)? [
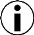
](http://www.uoguelph.ca/research/services-divisions/ethics/reb-app-guidelin#secdque)

Yes  NO

If **NO**, please go to D.6

If **YES** to proxy consent please answer the following**:**

D.5.1 Why is proxy consent necessary? [
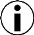
](http://www.uoguelph.ca/research/services-divisions/ethics/reb-app-guidelin#secdque0)

|  |
| --- |

D.5.2 How will competence of the participant be established, and who will determine this?

N/A [
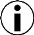
](http://www.uoguelph.ca/research/services-divisions/ethics/reb-app-guidelin#secdque0)

|  |
| --- |

D.5.3 Will you be obtaining informed assent from the participant?  Yes  No [
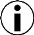
](http://www.uoguelph.ca/research/services-divisions/ethics/reb-app-guidelin#secdque0)

If **NO**, explain why not:

|  |
| --- |

D.5.4 How will oral assent, if used, be documented?  N/A [
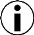
](http://www.uoguelph.ca/research/services-divisions/ethics/reb-app-guidelin#secdque0)

|  |
| --- |

D.5.5 Verify the following: [
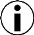
](http://www.uoguelph.ca/research/services-divisions/ethics/reb-app-guidelin#secdque0)

| Yes | N/A | Copy of written assent form attached to this application | Comment |
| --- | --- | --- | --- |
| Yes | N/A | Written assent form printed on University of Guelph letterhead | Comment |
| Yes | N/A | Copy of written assent form will be given to participant | Comment |
| Yes | N/A | Copy of oral assent script attached to this application | Comment |
| Yes | N/A | Copy of written information for participant providing oral assent attached to this application | Comment |

D.5.6 Attestation regarding Proxy Consent: Article 3.9 TCPS2 [
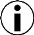
](http://www.uoguelph.ca/research/services-divisions/ethics/reb-app-guidelin#secdque0)

| Yes | N/A | the researcher will involve participants who lack the capacity to consent on their own behalf to the greatest extent possible in the decision-making process |
| --- | --- | --- |
| Yes | N/A | the researcher will seek and maintain consent from authorized third parties in accordance with the best interests of the persons concerned |
| Yes | N/A | the authorized third party is not the researcher or any other member of the research team |
| Yes | N/A | the research is being carried out for the participant’s direct benefit, or for the benefit of other persons in the same category. If the latter, the researcher has demonstrated that the research will expose the participant to only a minimal risk and minimal burden, and that the participant’s welfare will be protected throughout the participation in research |
| Yes | N/A | when authorization for participation was granted by an authorized third party, and a participant acquires or regains capacity during the course of the research, the researcher shall promptly seek the participant’s consent as a condition of continuing participation |

D.5.7 Are provisions planned for participants, or those consenting on a participant’s behalf, to have special assistance, if needed, during the consent process  Yes  No [
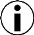
](http://www.uoguelph.ca/research/services-divisions/ethics/reb-app-guidelin#secdque0)

If **YES**, discuss:

|  |
| --- |

D.6 Deception: Are you using partial disclosure or deception (i.e. the participant may not know that they are part of a project until it is over or is not informed of the true purpose of the research in advance)?  Yes  No [
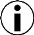
](http://www.uoguelph.ca/research/services-divisions/ethics/reb-app-guidelin#secdque)

If **NO** go to question D.8

If **YES** to deception or partial disclosure, please answer the following:

D.6.1 Describe the deception(s) or partial disclosure(s) being used and why they are necessary.

|  |
| --- |

D.6.2 Describe how and when the deception or partial disclosure will be revealed.

|  |
| --- |

D.6.3 State who will debrief the participants regarding the nature of the deception or partial disclosure, and describe how they have been trained. [
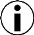
](http://www.uoguelph.ca/research/services-divisions/ethics/reb-app-guidelin#secdque0)

|  |
| --- |

D.6.4 Verify the following:

| Yes | N/A | A second consent form will be used. | Comment |
| --- | --- | --- | --- |
| Yes | N/A | Second consent form will be printed on University of Guelph letterhead | Comment |
| Yes | N/A | Copy of second consent form will be given to participant | Comment |
| Yes | N/A | If participant declines to sign the second consent form, data will be removed from the study without penalty. Participant will still receive any incentives or reimbursement due. | Comment |
| Yes | N/A | Copy of second consent form attached to this application | Comment |
| Yes | N/A | University of Guelph guideline on deception has been followed | Comment |

D.7 Is community or institutional consent required for your project?  Yes  No  N/A [
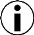
](http://www.uoguelph.ca/research/services-divisions/ethics/reb-app-guidelin#secdque)

Please discuss why this is required, how it will be managed.

|  |
| --- |

D.8 Will the participant be free to give consent, or refuse, without any undue influence or

coercion?  Yes  No [
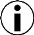
](http://www.uoguelph.ca/research/services-divisions/ethics/reb-app-guidelin#secdque)

Explain any details you feel are relevant.

| Subjects are free to remove themselves from the study at any point. The data will be deleted on their request |
| --- |

D.9 How will you ensure that consent is ongoing throughout the project? How will you ensure that necessary information is provided to participants on an ongoing basis?  N/A [
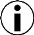
](http://www.uoguelph.ca/research/services-divisions/ethics/reb-app-guidelin#secdque)

| Participants will be told during each visit exactly what the procedure is going to be. They will also have blank copies of the original consent form, to which they can refer if necessary. |
| --- |

D. 10 Discuss the likelihood that the confidentiality offered to participants may be limited by the legal obligation to “report information to authorities to protect the health, life or safety of a participant or third party” or that “a third party may seek access to information obtained and/or created in confidence in a research context” through either “voluntary disclosure” or “force of law”.[TCPS2, Article 5.1]  N/A [
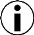
](http://www.uoguelph.ca/research/services-divisions/ethics/reb-app-guidelin#secdque0)

|  |
| --- |

## **D.11 Participant withdrawal** [
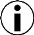
](http://www.uoguelph.ca/research/services-divisions/ethics/reb-app-guidelin#secdque0)

D.11.1 Participants must have the right to withdraw from the project at any time. Describe how the participants will be informed of their right to withdraw and outline the procedures that will be followed to allow the participants to exercise this right.

| In the Consent Form and during the first visit they will be informed of their right to withdraw from the study at any time. Subjects can refuse to sign the genomic consent form, yet remain in all other aspects of the study. |
| --- |

D.11.2 Indicate what will be done with the participants’ data and any consequences for the participant of withdrawing from the study. Participants must have the right to withdraw their data from the project. Exceptions include anonymous data and collectively recorded data (such as focus group recordings).

| There will be no consequece if the participant decides to remove themselves from the study. The blood samples will be disposed using appropriate biohazard procedures. |
| --- |

D.11.3 If the participants will not have the right to withdraw from the project, please explain.

|  |
| --- |

SECTION E: DESCRIPTION OF THE RISKS AND BENEFITS

E.1 Risks: Itemize your response by each method/procedure employed during this research. [
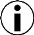
](http://www.uoguelph.ca/research/services-divisions/ethics/reb-app-guidelin#seceque)

Risk (check all that apply)

| N/A |  |
| --- | --- |
| Yes | Physical (including bodily contact or administration of any substance) |
| Yes | Psychological (including feeling demeaned, embarrassed, worried, or upset) |
| Yes | Social (possible loss of status or reputation) |
| Yes | Economic (risk to livelihood or income) |

E.1.1 If you indicated **YES** to any of the above, are any of the risks indicated greater than the participant would encounter in their everyday life?  Yes  No

E.1.2 For each risk identified above describe how the risk will be managed and include an

explanation as to why alternative approaches could not be used.  N/A [
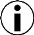
](http://www.uoguelph.ca/research/services-divisions/ethics/reb-app-guidelin#seceque0)

| Participants will have blood samples taken during the study, they may deal with minor bruises after blood samples. Participants will also be taking fish oil (omega 3) or a placebo oil supplement. Minor side effects may occur in some participants with fish oil supplementation, including belching, and bad breath. In rare cases, side effects may include heartburn, nausea, loose stools, rash, and nosebleeds. These minor side effects can be minimized if the supplement is kept frozen and taken with meals. Participants may also experience sensation of minor fatigue after VO2max test. |
| --- |

E.2 Possible Benefits Describe any benefits to the participants/discipline/ society that would

justify to participants why they should be involved in this study.  N/A [
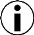
](http://www.uoguelph.ca/research/services-divisions/ethics/reb-app-guidelin#seceque)

| Benefits to Participant | The subjects will have their VO2 max and Resting Metabolic Rate measured, and will learn whether they respond to omega supplementation. We will provide individuals with details regarding their basic blood measurements (e.g. triglycerides, cholesterol, etc). They will also receive a small financial compensation. |
| --- | --- |
| Benefits to Discipline | Building upon current knowledge base on omega 3 supplementation and its effect on healthy young males. |
| Benefits to Society | These results may have large societal benefits if omega 3 supplementation proves beneficial for human metabolic health. |

E.2.1 Research results should be provided to participants where possible.

Will aggregate research results be provided to participants?  Yes  No [
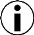
](http://www.uoguelph.ca/research/services-divisions/ethics/reb-app-guidelin#seceque0)

If **YES,** explain what information will be provided to the participants upon completion of the project, and how will they receive this.

| Results will be provided in a one page document (Feedback Form). They will receive this information upon completion of the study. |
| --- |

If **NO**, explain why this is not feasible or desirable.

|  |
| --- |

E.2.2 Will an individual’s research results be provided to participants?  Yes  No [
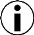
](http://www.uoguelph.ca/research/services-divisions/ethics/reb-app-guidelin#seceque0)

If **YES,** explain what information will be provided to the participants upon completion of the project, and how will they receive this.

| They will a one-page reports listing their results and receive a hard copy of the final manuscript. |
| --- |

SECTION F: CONFIDENTIALITY AND DATA SECURITY

F. 1 **Indicate what** type of information **will be collected** [
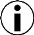
](http://www.uoguelph.ca/research/services-divisions/ethics/reb-app-guidelin#secfque)

| Yes | No | Directly Identifying Information |
| --- | --- | --- |
| Yes | No | Indirectly Identifying Information |
| Yes | No | Coded Information |
| Yes | No | Anonymized Information |
| Yes | No | Anonymous Information |

|  |
| --- |

F.2 Describe any personal identifiers – both direct and indirect - that will be collected during the course of the research and justify the need to collect them. Researchers should reduce the number of identifiers to a minimum. [
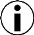
](http://www.uoguelph.ca/research/services-divisions/ethics/reb-app-guidelin#secfque)

| The subjects names, e-mail adresses and phone numbers will be initially collected. Then, all subjects will be given a code to ensure confidentiality. A master file linking participant IDs to individual subjects will be stored for five years in a locked filing cabinet and in a password protected file on researchers’ computers. |
| --- |

F.3 Under some circumstances, identified data must be made available to authorities. This may occur at the request of auditors (e.g. Health Canada, Tri-Council), or under subpoena. Describe the likelihood of this applying to this research project, and how or if you plan to communicate this possibility to participants.  N/A

|  |
| --- |

F.4 Describe any action that should be taken by the Principal Investigator prior to beginning the project, or any information which should be communicated to participants in the consent process, which deals with potential incidental findings.  N/A [
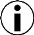
](http://www.uoguelph.ca/research/services-divisions/ethics/reb-app-guidelin#secfque)

| This study is for research purposes only and is results are not used for diagnostic purposes. All genetic markers examined will be related to fish oil supplementation and markers of cardiometbolic health. The results from genetic analyses of this study will not be shared with the participants, unless specifically requested. Upon this request, the participants will be asked to schedule a meeting with Dr. Mutch or one of his research students in order to discuss the results. This will allow for clear explanation of the results related to fish oil supplementation and markers of cardiometabolic health.  If there are cardiometabolic markers (such as triglycerides, cholesterol etc.) which have numbers well outside of the normal range, the participants will be notified and asked to speak with their physician for further information. |
| --- |

F.5 If any personal identifiers will be retained once data collection is complete, provide a comprehensive rationale explaining why it is necessary to retain this information – including the retention of master lists that link participant identifiers with unique study codes and de-identified data  N/A [
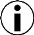
](http://www.uoguelph.ca/research/services-divisions/ethics/reb-app-guidelin#secfque)

| See F.2. |
| --- |

F.6 If existing records (e.g. health records, other records/databases) are to be used, describe how permission was obtained.  N/A [
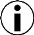
](http://www.uoguelph.ca/research/services-divisions/ethics/reb-app-guidelin#secfque)

Submit Appendix X – Secondary use of Data

|  |
| --- |

F.7 What would the impact be on the participant should privacy be breached? [
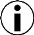
](http://www.uoguelph.ca/research/services-divisions/ethics/reb-app-guidelin#secfque)

| There is unlikely to be much impact on the participant should this data be released with identifiers. |
| --- |

F.8 State who else will have access to the identified data. If they are not members of the research team, they should sign a confidentiality agreement  N/A [
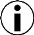
](http://www.uoguelph.ca/research/services-divisions/ethics/reb-app-guidelin#secfque)

|  |
| --- |

F.9 Describe the procedures to be used to protect the identity of the participant and/or ensure the security of the data: [
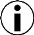
](http://www.uoguelph.ca/research/services-divisions/ethics/reb-app-guidelin#secfque)

F.9.1 During the conduct of the research:  N/A

| All subjects will be given a code to ensure confidentiallity. |
| --- |

F.9.2 During processing of data:  N/A

| Same as above |
| --- |

F.9.2.1 Will data be transcribed?  Yes  No [
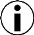
](http://www.uoguelph.ca/research/services-divisions/ethics/reb-app-guidelin#secfque09)

If **YES**, attach copy of transcriber confidentiality agreement (if transcriber is not part of the research team)  Attached

|  |
| --- |

F.9.2.2 Will the identified data be transferred electronically?  Yes  No

If **YES**, by what medium and how will it be protected during transit?

| Data will be stored in databases on the researchers’ personal computers for five years. These are three computers (Dr. Spriet’s, Dr. Mutch's, and Sebastian Jannas’), which will be encrypted to ensure complete confidentiality. Hard copies of data summaries will also be made and kept on file. All data will be coded by subject identification numbers (see above) in order to protect their confidentiality. |
| --- |

F.9.3 After research is complete:  N/A

| Same as above |
| --- |

F.9.4 In the release of findings:  N/A

| Same as above |
| --- |

## **F.10 Long Term Data Security** [
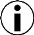
](http://www.uoguelph.ca/research/services-divisions/ethics/reb-app-guidelin#secfque0)

Discuss how long data will be stored, justify the duration of the storage period, discuss the security measures which will be employed, and name the individual who will be charged with stewardship of the data:

| Data will be kept for five years following completion of the study. Dr. Spriet and Dr. Mutch will be in charge of stewardship of data. |
| --- |

F.10.1 Will paper records be retained, and if so, which of the following apply?

Confidential shredding after 5 years.

De-identified data will be retained in secure location

Identified data will be retained in secure location

Describe secure location:

| It will be kept in a file under lock. |
| --- |

F.10.2  Will audio/video recordings be retained, and if so, which of the following apply?

Destruction of audio/video recordings after

Will be retained in secure location

Describe secure location:

|  |
| --- |

F.10.3  Will electronic data be retained, and if so, which of the following apply?

Secure erasing of electronic data after 3 years.

De-identified data will be retained in secure location

Identified data will be retained in secure location

Describe secure location:

| Data will be stored in databases on the researchers’ personal computers. These are three computers (Dr. Spriet’s, Dr. Mutch's, and Sebastian Jannas’), which will be encrypted to ensure complete confidentiality |
| --- |

F.11 Do you intend to link the data you have gathered with any other set of data?

Yes  No

Describe:

|  |
| --- |

Section G: Post Approval

## **G.1 Continuing Ethics Review** [
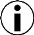
](http://www.uoguelph.ca/research/services-divisions/ethics/reb-app-guidelin#secgque)

Minimum requirement for Continuing Ethics Review is the submission of a Status Report at least annually. The principal investigator’s responsibility for the project must notify the REB using the Status Report when the project is completed, or if it is cancelled.

Indicate whether any additional monitoring or review would be appropriate for this project.

N/A

|  |
| --- |

## **G.2 Adverse Events** [
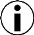
](http://www.uoguelph.ca/research/services-divisions/ethics/reb-app-guidelin#secgque)

Unanticipated consequences or results affecting participants must be reported to the Research Ethics Board and the Ethics Office as soon as possible using the <<adverse event report>>.

# G.3 Additional Information [
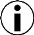
](http://www.uoguelph.ca/research/services-divisions/ethics/reb-app-guidelin#secgque)

Please add any other information relevant to the project that you wish to provide to the Research Ethics Board. N/A

|  |
| --- |

SECTION H: SIGNATURES

# PRINCIPAL INVESTIGATOR SIGNATURE:

I, ____________________________ [PLEASE PRINT] have the ultimate responsibility for the conduct of the study described in this application including my responsibilities as an advisor to any students involved in this project. I have read and am responsible for the content of this application. If any changes are made in the above arrangements or procedures, or adverse events are observed, I will bring these to the attention of the ETHICS OFFICE.

Signature Date

Graduate Student Signature (optional) Date

***NOTE: THIS PAGE IS REQUIRED IN HARD COPY BEARING THE SIGNATURE OF THE PRINCIPAL INVESTIGATOR***

***SEND TO THE ETHICS OFFICE:***

- ***HAND DELIVER OR BY UNIVERSITY MAIL to UC437***
- ***SCAN AND EMAIL TO*** [***reb@uoguelph.ca***](mailto:reb@uoguelph.ca)
- ***FAX TO 519.821.5236 and notify by email that fax has been sent.***
